# Incidence of Injuries Associated With Anterior Talofibular Ligament Injury Based on the Reporting of Magnetic Resonance Imaging

**DOI:** 10.7759/cureus.41738

**Published:** 2023-07-11

**Authors:** Don Koh, Darshana Chandrakumara, Charles Kon Kam King

**Affiliations:** 1 Orthopaedics, Changi General Hospital, Singapore, SGP; 2 Orthopaedic Surgery, Changi General Hospital, Singapore, SGP

**Keywords:** calcaneo-fibular ligament, deltoid ligament complex, anterior talofibular ligament, ligament injuries, chronic ankle instability, lateral ankle ligament instability, ankle instability, recurrent ankle sprain, ankle and foot, ankle mri

## Abstract

Introduction

This paper aims to report the incidence of ligamentous, tendon, and other structural injuries associated with an anterior talofibular ligament (ATFL) injury based on magnetic resonance imaging (MRI) findings.

Methods

The reports of all patients who underwent surgical treatment for ATFL injury between 2021 and 2022 at Changi General Hospital and had preoperative MRI ankle scans performed were analyzed in this retrospective study. Patients who had a preoperative MRI ankle scan performed with specific reporting of the ATFL, calcaneofibular ligament (CFL), deltoid ligaments, peroneal tendons, and the presence of an osteochondral defect (OCD) were included in this study. Patients who underwent surgery but did not have a preoperative MRI ankle scan done or had ankle fractures or systemic conditions affecting the same ankle were excluded.

Results

Eighty-six patients were included in this study, of which 59 were males and 27 were females. About 73.3% (63 of 86) of patients had sustained injuries in association with ATFL injury, and 58.1% (50 of 86) of patients suffered an associated injury to the calcaneofibular ligament (CFL). There were injuries to the superficial and deep deltoid ligaments in 29.1% (25 of 86) and 44.2% (38 of 86) of patients, respectively. The peroneal tendons were also injured in 17.4% (15 of 86) of patients. Lastly, there were also associated OCDs found in 19.8% (17 of 86) of patients.

Conclusion

There is a high incidence of injuries associated with an ATFL injury. The CFL and deltoid ligament complex are the most commonly injured structures in association with the ATFL. One in five patients will also have an associated OCD. The ATFL tends to be the only structure that is commonly addressed during surgery. Repair of the ATFL only may thus lead to poorer outcomes and persistent pain, if the underlying cause is due to the other concurrent injuries. Clinical evaluation of the other structures should thus be thoroughly performed to allow the addressing of any concurrent injuries in the same surgical setting to achieve better outcomes.

## Introduction

The incidence of ankle sprains is 19.0 to 26.6/1000 person-years in the general population [1,2] and even higher in the athletic population [3]. The ankle joint is a hinged synovial joint composed of the articulation between the tibia, fibula, and talus bone, which is stabilized by the medial and lateral ligament complexes as well as the syndesmotic ligaments. Ankle sprains result from either an eversion or inversion mechanism of injury, with the latter accounting for the majority of all ankle sprains. As a result, the lateral ankle ligament complex that resists inversion is the most commonly affected structure in an ankle sprain.

The lateral ankle ligament complex consists of the anterior talofibular ligament (ATFL), calcaneofibular ligament (CFL), and posterior talofibular ligament (PTFL). Originating from the anterior aspect of the distal fibula, the ATFL inserts onto the anterolateral surface of the talus to perform its primary function of resisting plantarflexion and inversion. The ATFL is the shortest and the weakest of the three ligaments, having only little capacity to maintain maximal tension until failure [4,5]. Therefore during an inversion ankle sprain, the ATFL is most commonly injured with involvement of up to 85% of all ankle sprains [6,7]. Comparatively, the CFL and PTFL have been reported to have much lower rates of involvement, from 35% to 75% and up to 12%, respectively [8].

The majority of ankle sprains are treated conservatively with good outcomes. However, complications such as chronic lateral ankle instability and pain have been reported in up to 25%-40% [9]. In this group of patients, further evaluation and imaging, most commonly with an MRI ankle, is done to rule out any injury to the ATFL and other structures.

Therefore, this article aims to describe the incidence of injuries sustained in association with an injury to the ATFL based on magnetic resonance imaging (MRI) findings.

## Materials and methods

A retrospective study was carried out by analyzing the MRI ankle reports of all patients who underwent surgical management for ATFL injury between 2021 and 2022 at Changi General Hospital. The three authors analyzed the MRI ankle reports with predetermined terms to interpret the reports for the classification of the integrity of each structure. The terms “scarred” or “scarring” were reported as sprain for the purposes of our study to reduce the heterogeneity of the terms used by reporting radiologists. In the event of a discrepancy over the interpretation of the MRI reports, the three authors would convene and come to a mutual agreement.

The inclusion criteria are having a preoperative MRI ankle scan of the affected ankle and the MRI ankle reporting specifically about the condition of ATFL, CFL, deltoid ligaments, peroneal tendons, and the presence of an osteochondral defect (OCD). Patients who underwent surgery for ATFL injury but did not have a preoperative MRI ankle scan performed were not included in the study. Patients who also had sustained fractures or systemic pathologies affecting the same ankle were excluded from the study.

This study was approved by the SingHealth IRB Institution, and the approval number is 2017/2947.

## Results

Patients

A total of 86 patients were included in this study, of which 59 were males and 27 were females. There were 41 left ankles and 45 right ankles in terms of laterality. The average age at the time of surgery was 34.6 years (range: 17-66 years). The aforementioned information is summarized in Table 1.

**Table 1:** Patient demographics

Measurement	Value
No. of patients	86
Age (Years)	34.6 ± 13.8 (range: 17–66 years)
Gender	Male: 59 (68.6%)
	Female: 27 (31.4%)
Body mass index (kg/m^2^)	30.58 ± 5.6 (range: 18.1–40.2 kg/m^2^)
Operated side	Right: 45 (52.3%)
	Left: 41 (47.7%)

ATFL injury

**Figure 1:**
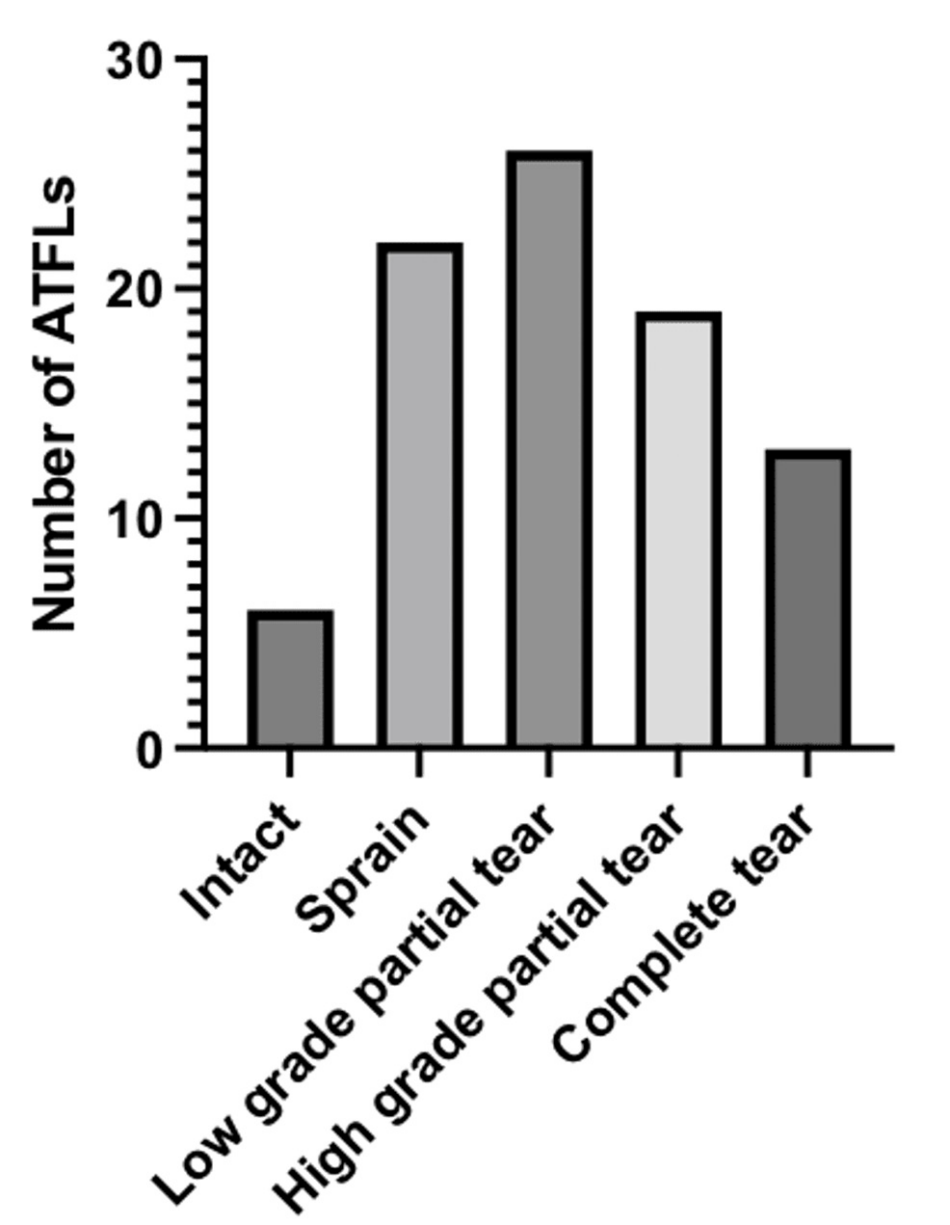
Extent of anterior talofibular ligament (ATFL) injury

The ATFL was reported to be intact in 7% (6 of 86) of patients, sprained in 25.6% (22 of 86), and torn in 67.4% (58 of 86). There were low-grade partial tears in 30.2% (26 of 86) of patients, and high-grade partial tears in 22.1% (19 of 86) of patients. The remaining 15.1% (13 of 86) of patients sustained complete tears of the ATFL. This information is shown in Figure 1 and summarized in Table 2.

**Table 2:** Extent of injuries and nature of injuries to the ATFL ATFL: Anterior talofibular ligament.

Measurement		Number (%)
Extent of injuries	Isolated injury	23 (26.7%)
	Associated injuries	63 (73.3%)
Anterior talofibular ligament (ATFL) integrity	Intact	6 (7.0%)
	Sprained	22 (25.6%)
	Low-grade partial tear	26 (30.2%)
	High-grade partial tear	19 (22.1%)
	Complete tear	13 (15.1%)

Associated injuries

**Figure 2:**
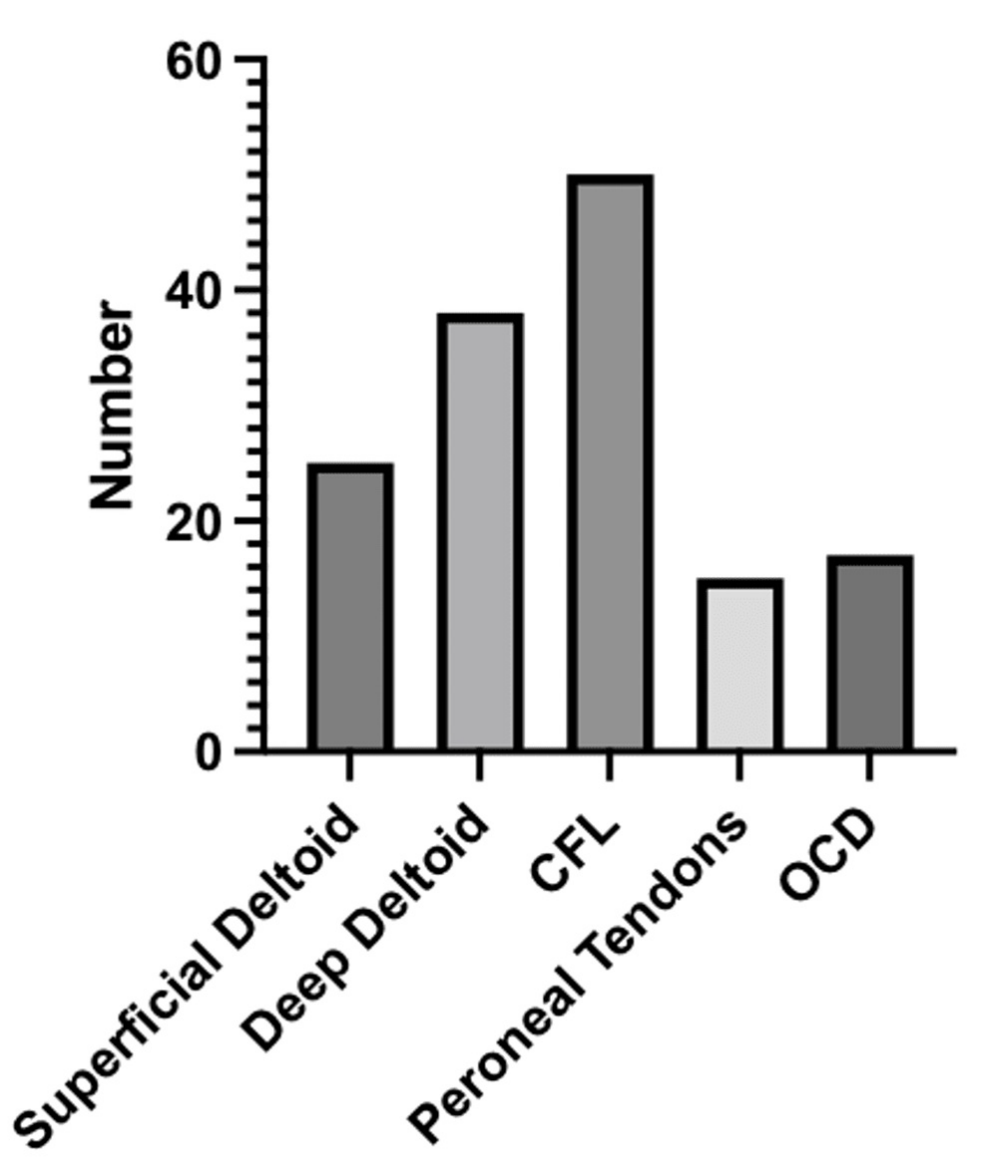
Associated injuries CFL: Calcaneofibular ligament; OCD: Osteochondral defect.

About 73.3% (63 of 86) of patients had sustained injuries in association with an ATFL injury, and 58.1% (50 of 86) of patients suffered injury to the CFL. There were injuries to the superficial and deep deltoid ligaments in 29.1% (25 of 86) and 44.2% (38 of 86) of patients, respectively. The peroneal tendons were also injured in 17.4% (15 of 86) of patients. Lastly, associated OCDs were found in 19.8% (17 of 86) of patients. The aforementioned information is shown in Figure 2 and summarized in Table 3.

**Table 3:** Summary of associated injuries

Associated injury	Integrity	Number (%)
Calcaneofibular ligament (CFL)	Intact	36 (41.9%)
	Sprain	23 (26.7%)
	Low-grade partial tear	17 (19.7%)
	High-grade partial tear	6 (7.0%)
	Complete tear	4 (4.7%)
Deep deltoid	Intact	48 (55.8%)
	Sprain	21 (24.4%)
	Low-grade partial tear	13 (15.1%)
	High-grade partial tear	4 (4.7%)
Superficial deltoid	Intact	61 (70.9%)
	Sprain	11 (12.8%)
	Low-grade partial tear	11 (12.8%)
	High-grade partial tear	3 (3.5%)
Peroneal tendon	Intact	71 (82.6%)
	Tear	4 (4.6%)
	Tendinosis	11 (12.8%)
Osteochondral defect (OCD)	Associated OCD	17 (19.8%)
	No associated OCD	69 (80.2%)

## Discussion

Calcaneofibular ligament (CFL)

The CFL was noted to be the most commonly injured ligament (58.1%) in association with an ATFL injury, in keeping with existing knowledge [10]. The importance of the CFL in providing lateral stabilization and preventing inversion of the ankle and subtalar joint has been shown in multiple studies [11,12], and the high incidence of its injury in association with ATFL injury from an inversion ankle sprain is therefore not surprising. MRI sensitivity for CFL injury has been reported to vary based on the extent of injury to the CFL sustained [13]. Accuracies as low as 66% and 88% have been reported for partial and complete tears of the CFL [14]. Sensitivity has also been reported to be much lower in the setting of chronic CFL injuries [15]. Given the high incidence of associated CFL injuries, clinical suspicion should remain high even in the setting of an ATFL injury as it may be missed on radiographic imaging. The medial talar tilt stress test is one such tool, which has been shown to isolate the CFL in cadaveric studies [16]; thus, if done properly, it can aid in the clinical diagnosis of injury to the CFL even if it is reported as normal on MRI.

Deltoid ligament

The medial deltoid ligament is the strongest of the ankle ligaments and consists of both the superficial deltoid and the deep deltoid ligament. The superficial deltoid stabilizes the function of the medial ligaments by limiting talar abduction, while the larger deep deltoid limits the external rotation of the talus on the distal tibia and provides more support [17,18]. Injury to the deltoid ligaments, therefore, arises in an eversion ankle sprain and occurs rarely in isolation [19].

The incidence of injury to the deep and superficial deltoid ligament in association with an ATFL injury was 44.2 and 29.1%, respectively. Current evidence also suggests that the deltoid ligament is involved in up to 15% of ankle inversion injuries [20], more than what was previously thought. Careful attention should thus be given to evaluating the medial deltoid ligament complex, even in the setting of a lateral ankle injury. Appropriate and timely treatment for these associated injuries can prevent the development of medial ankle instability and its subsequent complications.

Peroneal tendons

The peroneus longus tendon was reported to play a role as a stabilizer in lateral ankle sprains based on data analyzed by Ziai et al. [21]. An incidence injury to the peroneal tendons of 17.4% in association with an ATFL injury as seen in our study is therefore not surprising. Previous studies have also shown the underrecognition of peroneal tendon tears after ankle sprains [22]. The peroneal tendons should be evaluated alongside the ATFL and surgically addressed in the same setting if required [23].

Osteochondral defects (OCDs)

OCDs are talus injuries that range from superficial cartilage damage to cartilage fractures, resulting in pain.Trauma is generally accepted as the primary etiology of OCD [24], a large proportion of which is made up of ankle sprains. Forced rotation of the talus within the mortise during ankle sprains results in direct damage to the cartilage [25]. Cadaver studies have also reproduced this finding, which displays the direct compression and shearing of the lateral aspect of the talus against the articular aspect of the fibula during an inversion injury, resulting in lateral OCD [26]. OCDs have also been reported to have an association with chronic lateral ankle instability as a result of ATFL injury [27,28], with the proposed mechanism being repetitive microtrauma or misalignment [25]. The incidence of 19.8% for OCDs in association with ATFL injury seen in our study is thus in keeping with existing knowledge.

Limitations

Our study has some limitations. First, the study only allows us to state associations in the South East Asian population. A similar study reporting structural injuries associated with injury to the ATFL based on MRI was performed in the Spanish population [29], to the author’s knowledge; however, comparative studies between various ethnic groups are still lacking. Second, intra-observer and inter-observer variability of MRI ankle reporting was not checked in the course of our study. A previous study had reported low inter-observer variability in US ankle reporting, with findings comparable to MRI [30]; however, data specific to MRI ankle reporting is still lacking. Third, the timing of the MRI ankle being performed with regard to the chronicity of the injury was not controlled, which may affect the reporting of injuries.

## Conclusions

There is a high incidence of injuries associated with an ATFL injury. The CFL and deltoid ligament complex are the most commonly injured structures in association with the ATFL. One in five patients will also have an associated OCD. The ATFL tends to be the only structure that is commonly addressed during surgery. Repair of the ATFL only may thus lead to poorer outcomes and persistent pain, if the underlying cause is due to the other concurrent injuries. Surgical repair of the deltoid ligament complex, peroneal tendons, and treatment of the OCD, if indicated, should be addressed in the same initial setting of ATFL repair to prevent the need for multiple surgeries and to ensure the best outcomes.
